# Evaluation of diode laser efficacy in treating benign oral soft tissue masses: A case series

**DOI:** 10.1016/j.ijscr.2023.109075

**Published:** 2023-11-20

**Authors:** Irna Sufiawati, Fitri Dona Siregar, Indah Suasani Wahyuni, Endang Syamsudin

**Affiliations:** a Department of Oral Medicine, Faculty of Dentistry, Universitas Padjadjaran, Bandung, Indonesia; b Oral Medicine Residency Program, Faculty of Dentistry, Universitas Padjadjaran, Bandung, Indonesia; c Department of Oral and Maxilofacial Surgery, Faculty of Dentistry, Universitas Padjadjaran, Bandung, Indonesia

**Keywords:** Benign tumor, Biopsy, Diode laser, Excision, Oral mucosa

## Abstract

**Introduction and importance:**

The diode laser, with a wavelength ranging from 810 to 980 nm, is a modern treatment modality that offers significant advantages in the management of benign oral soft tissue masses. Therefore, this report aimed to assess the efficacy of diode laser application for excisional biopsy of such masses.

**Case presentation:**

Three female patients, aged 9, 39, and 45 years, visited the Oral Medicine Clinic with complaints of painless masses in the oral cavity persisting for two to three months. Their intraoral examination showed the presence of pedunculated or sessile exophytic lesions with a smooth surface. The lesions appeared as single, pink to red protrusions located in various sites, including the left buccal mucosa, right lateral border of the tongue, and lower gingiva.

**Clinical discussion:**

Excisional biopsy of the masses was carried out in the patients using the diode laser. The surgical procedures ranged from 10 to 20 min, with minimal intraoperative bleeding and precise cutting, while no pain was reported. Postoperatively, at two weeks and four months follow-up, the intraoral wounds exhibited excellent healing without complications such as pain, bleeding, swelling, scarring, infection, or mass recurrence. The clinical diagnosis of irritational fibroma (two cases) and fibrous epulis were confirmed by histopathological examination.

**Conclusion:**

Diode laser emerges as a highly efficacious method for the excisional biopsy of benign oral soft tissue masses, providing intraoperative and postoperative advantages over scalpel surgery.

## Highlights

• The diode laser with wavelengths ranging from 810 to 980 nm, is beneficial for managing benign oral soft tissue masses.
• A diode laser used in oral mucosa surgery reduces swelling, bleeding, scarring, pain, and accelerates the healing process.
• The diode laser's compact, portable, cost-effective design make it ideal for various surgical applications in dentistry.

## 1 Introduction

The oral cavity is one of the most common sites for the occurrence of masses, which can be classified as odontogenic and non-odontogenic lesions. Several factors, including poor oral hygiene, removable dentures, smoking, malposition, bad habits, and mechanical irritation, influence the development of these masses. Oral lesions are often categorized into non-neoplastic, neoplastic, and potentially malignant groups. Meanwhile, the five groups provided by The International Classification of Diseases for Dentistry and Stomatology (ICD-DA) and the World Health Organization are reactive lesions, pre-cancerous/potentially malignant lesions, salivary gland pathology, benign masses, and malignant masses [[1], [2], [3], [4], [5]].

Benign masses do not metastasize but may cause local damage. They exhibit slow growth, lack invasiveness, possess low mitosis and high cell differentiation rates, and tend to recur [1,5]. These masses are more dominant in males (70.2 %), aligning with findings from Permi et al. (63.7 %) and Modi et al. (60.5 %) [6,7]. In the study by Ghai et al., a male-to-female ratio of 1:1 was documented. The buccal mucosa (50.6 %) is the most prevalent location for benign masses, although Agarwal et al., Modi et al., Kosam et al. stated gingiva as the commonest site [5,[7], [8], [9]].

A biopsy is suggested for oral lesions that cannot be diagnosed clinically or radiographically, but it should be avoided in cases of bleeding disorders. An excisional biopsy involves the complete removal of the lesion, which is then sent to the Anatomical Pathology department for histopathological examination. This technique is suitable for small clinically benign lesions. Either scalpel, punch, laser, or electrosurgical biopsy can be employed for oral lesion excision. Even though simple scalpel biopsy is routinely used due to being affordable, uncomplicated, accurate, and characterized by minimal damage to surrounding tissues, it does not provide good hemostasis in highly vascular or maxillofacial areas. Injection or diode laser as an adjunct to soft tissue surgical operation in the oral mucosa has been reported to minimize swelling, bleeding, scar tissue formation, and postoperative pain, along with the acceleration of wound healing [1,[10], [11], [12]].

The diode laser is a modern treatment modality introduced in dentistry in 1999. Furthermore, it is a solid-state semiconductor, which primarily uses a combination of gallium (Ga), arsenide (Ar), and other elements such as aluminum (Al) and indium (In), with wavelengths ranging from 810 to 980 nm. Prior studies have indicated that this device effectively removes premalignant lesions during the management of oral mucosa and maxillofacial diseases [11,[13], [14], [15]]. Both high-powered (hot, hard) and low-powered (cold, soft) diode lasers can be employed for the treatment of benign masses such as irritation fibroma and fibrous epulis [12,15]. This study aimed to evaluate the efficacy of diode laser application for excision biopsy of benign soft tissue masses in the oral cavity.

We performed a prospective single-center consecutive case series with the inclusion criteria involving patients with oral benign masses who underwent treatment using a diode laser. The study was conducted at Oral Medicine, Teaching General Hospital of Padjadjaran University. All patients were operated on by an experienced oral medicine specialist and oral surgeon. This case series has been reported in line with the PROCESS 2020 (www.processguideline.com) criteria [16].

## 2 Case presentation

### 2.1 First case

A 9-year-old female patient presented to the Oral Medicine department with a complaint of a mass on the lateral right side of her tongue, causing discomfort without pain for the past two months. He had no previous history of surgery, smoking, recurrent mouth ulcers, fever, systemic diseases, and use of recreational drugs or alcohol abuse.

**Fig. 1:**
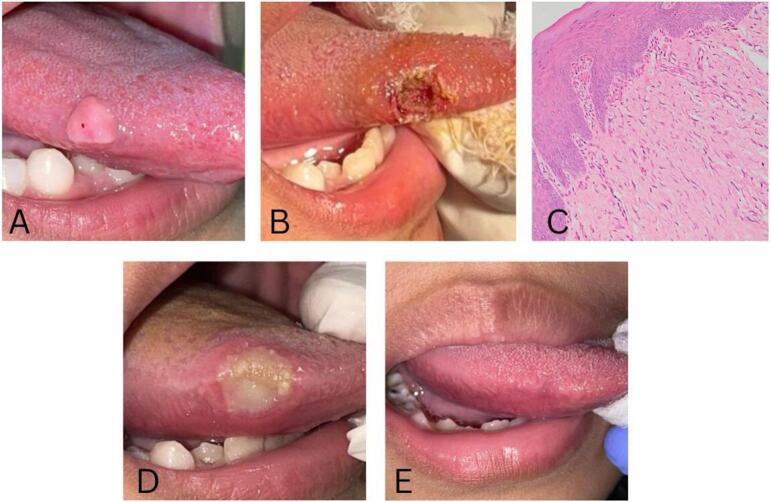
Clinical presentation of irritation fibroma in a 9-year-old female patient treated with a diode laser. (A) Tumor measuring 0.8 × 0.6 × 0.6 cm on the lateral right side of the tongue; (B) Post-excision biopsy using a diode laser technique; (C) Histopathological examination revealed hyperkeratotic squamous epithelium, dense collagen stroma, packed spindle-shaped fibroblasts with collagen and areas of hyalinization, minimal inflammatory cells and vascularity (magnification at 100×). (D) A healing ulcer, 1 cm in diameter, was observed on the right lateral border of the tongue after two weeks of treatment; (E) The lesion completely healed after six weeks of treatment.

Intraoral examination revealed a single, smooth-surfaced, pink, pedunculated, or sessile exophytic lesion measuring 0.8 × 0.6 × 0.6 cm, located on the lateral right side of the tongue (Fig. 1A). Based on the clinical examination, the patient was diagnosed with irritation fibroma. An excisional biopsy of the mass using a diode laser technique was performed. The surgery lasted for around 15 min with minimal intraoperative bleeding and precise cutting, while no pain was experienced. The provided post-operative pharmacological therapy included three times daily administration of 250 mg tablets of amoxicillin and paracetamol for five and three days, respectively, as well as topical application of 0.1 % triamcinolone acetonide in oral base to the surgical site (Fig. 1B). The excised mass was placed in a formalin solution and sent to the Department of Anatomical Pathology for diagnosis, confirming it as fibroma. Histopathological examination showed nonencapsulated nodular mass, hyperkeratotic surface, squamous epithelium encasing the mass in dense collagen stroma. The stroma comprised tightly-packed spindle-shaped fibroblasts, with collagen arranged in parallel or dense patterns and areas of hyalinization, also scattered chronic inflammatory cells and minimal vascularity was observed (Fig. 1C).

During the second visit, two weeks after the treatment, the patient did not report any pain in her oral cavity and consistently followed the therapy administered by the oral medicine specialist. Intraoral examination revealed a single, round-shaped ulcer with irregular borders, covered by a yellowish-white pseudomembrane and surrounded by diffuse erythema, measuring 1 cm in diameter on the lateral right side of the tongue (Fig. 1D). After six weeks of treatment, the oral lesion disappeared, healing excellently with no complications such as pain, bleeding, swelling, scarring, infection, or mass recurrence (Fig. 1E).

### 2.2 Second case

A 39-year-old female patient presented to the Department of Oral Medicine with a chief complaint of a painless mass on the gingiva, which was persistent for two and a half months, causing discomfort. He had no prior history of smoking, recurrent mouth ulcers, fever, systemic diseases, and use of recreational drugs or alcohol abuse. However, he did report a previous history of a mass that was present five years ago and surgically removed through conventional means.

**Fig. 2:**
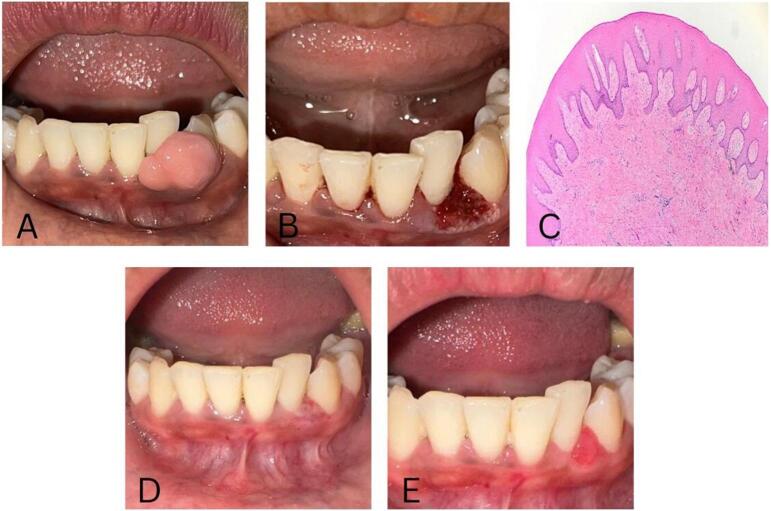
Removal of fibrous epulis on the gingiva in a 39-year-old female by diode laser. (A) Tumor measuring 1 × 0.8 × 0.5 cm on the left lower gingiva; (B) Post-excision biopsy with the diode laser technique; (C) Histopathological examination revealed hyperplastic squamous epithelium, fibrous tissue, inflammatory cell infiltration, capillary blood vessel proliferation, and some hemorrhage in the fibrous epulis, with no evidence of malignancy (magnification at 20×); (D) Clinical presentation at the second visit, two weeks after treatment, showing a healing ulcer covered with a pseudomembrane at the site of the excised tumor; (E) The lesion disappeared completely after four weeks of treatment.

Intraoral examination revealed a single pedunculated or sessile exophytic lesion with a smooth surface. The lesion appeared pink in color, measuring 1 × 0.8 × 0.5 cm, and located on the gingiva in the region of teeth 32–33. (Fig. 2A). An excisional biopsy of the mass using a diode laser technique was performed by the oral medicine specialist. The surgery took around 15 min, with minimal intraoperative bleeding and precise cutting, and the patient did not report any pain. The postoperative pharmacological therapy given to the patient included a regimen of 500 mg amoxicillin tablets and paracetamol, administered three times daily for five and three days, respectively. Subsequently, 0.1 % triamcinolone acetonide in an oral base was topically applied to the surgical site (Fig. 2B). The removed mass was placed in a formalin solution tube and dispatched to the Department of Anatomical Pathology for examination, which subsequently confirmed it as a fibrous epulis. Histopathological examination showed hyperplastic squamous epithelium, along with fibrous tissue that included the proliferation of capillary blood vessels and some hemorrhage. Inflammatory cells such as neutrophils, lymphocytes, histiocytes, and plasma cells infiltrated the stroma, without evidence of malignancy (Fig. 2C).

At the second visit after two weeks of treatment, the patient did not report any pain from the oral cavity and consistently followed the administered therapy. Intraorally, a white-yellowish ulcer covered with a pseudomembrane measuring 0.1 × 0.1 cm on the gingiva was observed (Fig. 2D). At the final visit, four weeks following the treatment, the gingival lesion had fully disappeared, showing excellent healing with no reported complications, including pain, bleeding, swelling, scarring, infection, or a recurrence of the mass (Fig. 2 E).

### 2.3 Third case

A 45-year-old female patient presented to the Department of Oral Medicine with a complaint of a painless mass in the left buccal mucosa that had been persistent for two months, causing discomfort. He had no previous history of surgery, smoking, recurrent mouth ulcers, fever, systemic diseases, and use of recreational drugs or alcohol abuse.

**Fig. 3:**
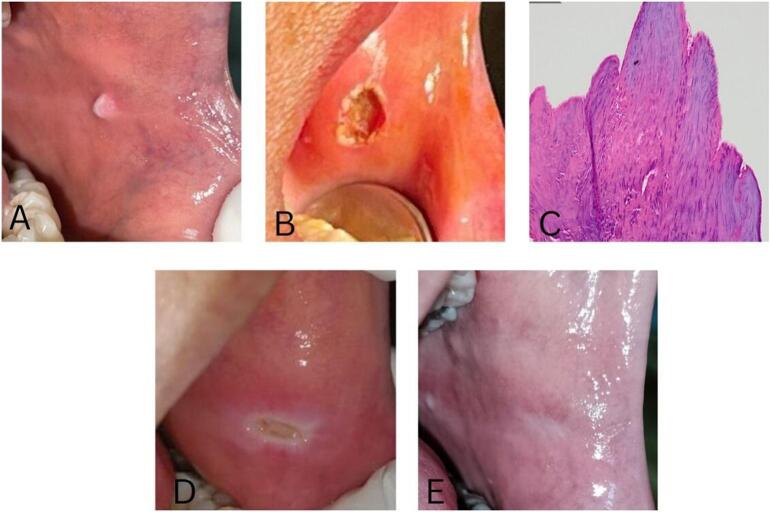
Clinical presentation of fibroma in a 45-year-old female patient. (A) A tumor measuring 0.6 × 0.5 × 0.3 cm on the left buccal mucosa; (B) The lesions after excision biopsy with a diode laser; (C) Histopathological appearance showed hyperkeratosis and the mass within a dense collagen stroma, hyaline degenerated fibro-collagen connective tissue, minimal inflammatory cells and vascularity (magnification at 100×). D) An ulcer measuring 0.2 × 0.1 cm on the left buccal mucosa after 14 days of treatment; (E) The lesion completely healed within four weeks of treatment.

Intraoral examination revealed a smooth-surfaced, pedunculated, or sessile, solitary exophytic lesion measuring 0.6 × 0.5 × 0.3 cm, with a pink color, was observed in the left buccal mucosa. (Fig. 3A). Based on the clinical examination, the patient was diagnosed with an irritation fibroma. A laser diode excision biopsy, lasting approximately 15 min, was carried out with minimal intraoperative bleeding, precise incisions, and no reported pain complaints. The provided postoperative pharmacological therapy included three times daily administration of 500 mg tablets of amoxicillin and paracetamol for five and three days, respectively, followed by the application of 0.1 % triamcinolone acetonide in oral base to the surgical site (Fig. 3B). The excised mass was transferred to a tube filled with formalin solution and sent to the Department of Anatomical Pathology for examination, which subsequently confirmed it as fibroma. Histopathological examination showed nonencapsulated nodular mass, hyperkeratotic surface, squamous epithelium encasing the mass in dense collagen stroma. The stroma comprised tightly-packed spindle-shaped fibroblasts, with collagen arranged in parallel or dense patterns and areas of hyalinization, also scattered chronic inflammatory cells and minimal vascularity was observed (Fig. 3C).

During the follow-up period of 14 days, the patient did not complain of any pain in her oral cavity and consistently followed the administered therapy. Intraoral examination revealed a white-yellowish ulcer covered with a pseudomembrane surrounded by diffuse erythema, measuring 0.2 × 0.1 cm on the left buccal mucosa (Fig. 3D). At the last visit, after four weeks of treatment, the oral mucosal lesion disappeared, indicating excellent healing without complications such as pain, bleeding, swelling, scar tissue, infection, or recurrence of the mass (Fig. 3E).

## 3 Discussion

A diode laser was used to perform excision biopsies on benign masses in all patients. This article presents a case series review, evaluating the efficacy of diode laser treatment for benign oral soft tissue masses, with the patients' consent already obtained. Diode laser radiation proved to be an excellent, simple, and safe method for treating oral lesions. This offers several advantages over conventional surgical techniques, including minimal damage to surrounding tissues, improved visibility, quick operation time, reduced pain, enhanced accuracy in treating soft tissue lesions, minimal scar tissue formation, and preservation of tissue elasticity [11]. The patients in this report expressed satisfaction with the laser surgery, as they experienced no pain during or after the operation. The diode laser operated through photokinetic, thermal effects, or plasma-mediated mechanisms, leading to the ablation or decomposition of biological materials. This device has been approved by the Food and Drug Administration for the therapy of various soft tissue conditions, including soft tissue curettage, incision, pocket debridement, and ablative excision [11,12].

The diode device was characterized by a small size appearance, portability, and relatively lower cost compared to other laser equipment. These features make it an attractive choice for dentists and specialists in various surgical indications. The diode laser could be used at three wavelengths, namely 810, 940, and 980 nm. A prior study investigated the thermal tissue effects of diode lasers with wavelengths of 830 nm and 940 nm in contact applications, reporting low thermal effects at the carbonization zone on the surface of histological tissue examination [17]. Specialists have affirmed the efficacy of the diode laser as an alternative device for soft tissue surgery in the oral cavity compared to other types, such as carbon dioxide (CO_2_) and erbium lasers [12,13].

In this report, a diode laser with a 976 nm wavelength, a 400 μm diameter tip, a pulse repetition rate of up to 50 kHz, a pulse duration of 2 milliseconds, and a power range of 2.0–10.0 W (Solase®) was used. This low-power wavelength was chosen to minimize postoperative complications such as burning and pain, as observed in our three cases [[11], [12], [13], [14], [15]]. Our experience aligns with prior research exploring various laser types for oral mucosal surgery, and we achieved successful laser therapy for these three cases, resulting in satisfactory wound healing, consistent with previously published findings.

The postoperative pharmacological therapy following laser excision biopsy included 250 mg tablets of amoxicillin and paracetamol administered three times daily for five and three days, respectively, along with 0.1 % triamcinolone acetonide in an oral base applied to the surgical site. Amoxicillin was prescribed to ensure proper wound management, promote healing, and prevent local infection by the polymicrobial target microbiota that could arise from surgical contamination or infection by normal oral and saliva microbiota. Natarajan et al., reported that amoxicillin has been commonly used as the first-choice antibiotic by dental practitioners in post-surgical scenarios [18]. Paracetamol was indicated as an analgetic [19], while 0.1 % triamcinolone acetonide dental paste (USP) was applied to eliminate inflammation and ulcerative lesions resulting from trauma to the oral mucosa post-surgery [20].

During the follow-up for two weeks and four months after the last visit, all patients reported no complaints after the surgery. Moreover, they were educated about maintaining oral hygiene, caring for their removable dentures, avoiding smoking and bad habits, and preventing mechanical irritation to minimize the risk of mass recurrence in the future.

## 4 Conclusion

Diode laser emerges as a highly efficacious method for the excisional biopsy of benign oral soft tissue masses, providing intraoperative and postoperative advantages over scalpel surgery.

## Informed consent

For case 1, written informed consent was obtained from the patient's parents for publication and any accompanying images. For cases 2 and 3, written informed consent was obtained from the patient for publication and any accompanying images. A copy of the written consent is available for review by the Editor-in-Chief of this journal on request.

## Ethical approval

This study is exempt from ethical approval in our institution for publishing anonymous case reports/case series.

## Funding

The authors thank Universitas Padjadjaran for funding the publication.

## Author contribution

Irna Sufiawati: Conception, design of the study, acquisition of the data, drafting and revising of the manuscript, final approval of the version to be submitted, and involved in direct management of the patient.

Fitri Dona Siregar: Acquisition of the data, drafting and revising of the manuscript, final approval of the version to be submitted, and also as the assistant in management of the patients.

Endang Syamsudin: Acquisition of the data, drafting the manuscript, final approval of the version to be submitted, and also involved in direct management of the patient.

Indah Suasani Wahyuni: Acquisition of the data, final approval of the version to be submitted.

All authors agreed to be accountable for all aspects of the manuscript.

## Guarantor

Irna Sufiawati.

## Research registration number

Not applicable.

## Declaration of competing interest

There are no conflicts of interest.

## Data Availability

The authors of this manuscript are willing to provide any additional information regarding the case report upon official request.
